# Binding Studies of AICAR and Human Serum Albumin by Spectroscopic, Theoretical, and Computational Methodologies

**DOI:** 10.3390/molecules25225410

**Published:** 2020-11-19

**Authors:** Shokoufeh Hashempour, Nahid Shahabadi, Aishat Adewoye, Brennen Murphy, Camaray Rouse, Brian A. Salvatore, Christopher Stratton, Elahe Mahdavian

**Affiliations:** 1 Department of Chemistry and Physics, Louisiana State University, Shreveport, LA 71115, USA; s.a_hashempour@yahoo.com (S.H.); adewoyea07@lsus.edu (A.A.); MURPHYB30@lsus.edu (B.M.); rousec04@lsus.edu (C.R.); bsalvato@lsus.edu (B.A.S.); strattonc68@lsus.edu (C.S.); 2 Department of Inorganic Chemistry, Faculty of Chemistry, Razi University, Kermanshah 6714414971, Iran; NahidShahabadi@yahoo.com; 3 Medical Biology Research Center (MBRC), Kermanshah University of Medical Sciences, Kermanshah 6714414971, Iran

**Keywords:** AICAR, human serum albumin (HSA), static fluorescence quenching, synchronous fluorescence, 3D fluorescence, FRET, Trp214, fluorophore microenvironment, molecular docking, MOE

## Abstract

The interactions of small molecule drugs with plasma serum albumin are important because of the influence of such interactions on the pharmacokinetics of these therapeutic agents. 5-Aminoimidazole-4-carboxamide ribonucleoside (AICAR) is one such drug candidate that has recently gained attention for its promising clinical applications as an anti-cancer agent. This study sheds light upon key aspects of AICAR’s pharmacokinetics, which are not well understood. We performed in-depth experimental and computational binding analyses of AICAR with human serum albumin (HSA) under simulated biochemical conditions, using ligand-dependent fluorescence sensitivity of HSA. This allowed us to characterize the strength and modes of binding, mechanism of fluorescence quenching, validation of FRET, and intermolecular interactions for the AICAR–HSA complexes. We determined that AICAR and HSA form two stable low-energy complexes, leading to conformational changes and quenching of protein fluorescence. Stern–Volmer analysis of the fluorescence data also revealed a collision-independent static mechanism for fluorescence quenching upon formation of the AICAR–HSA complex. Ligand-competitive displacement experiments, using known site-specific ligands for HSA’s binding sites (I, II, and III) suggest that AICAR is capable of binding to both HSA site I (warfarin binding site, subdomain IIA) and site II (flufenamic acid binding site, subdomain IIIA). Computational molecular docking experiments corroborated these site-competitive experiments, revealing key hydrogen bonding interactions involved in stabilization of both AICAR–HSA complexes, reaffirming that AICAR binds to both site I and site II.

## 1. Introduction

Human serum albumin (HSA) is the most abundant blood serum protein [1]. It transports and interacts with many endogenous substances (water, small cations, fatty acids, hormones, bilirubin) and exogenous ligands, including small bioactive drugs [1,2]. HSA is an ideal drug-delivery and transport protein, mainly because of its extraordinary ligand binding properties, excellent water solubility, and stability profiles (pH range of 4–9 and temperature up to 60 °C for 10 h) [2,3]. Most orally administered drugs are hydrophobic and poorly soluble in blood plasma, thus drug–HSA complexes aid in the systemic drug’s solubility and distribution [1,2,3]. Binding to HSA also improves drug stability, half-life, and differential uptake into diseased cells vs. the normal cells [4]. Therefore, HSA is often used as an appropriate in vitro model transport protein for studying the structural and biochemical basis of drug–protein interactions and the evaluation of drug-like properties related to distribution, bioavailability, and efficacy [1].

HSA is a small globular protein (MW 66.7 kDa) comprising a single non-glycosylated polypeptide chain of 585 amino acids with a heart-shaped tertiary structure (80 Å × 80 Å × 30 Å) [5]. X-ray 3D structural analysis of HSA reveals three homologous α-helical domains (I–III), each including two subdomains, A and B, stabilized by a total of 16 disulfide bridges [5]. Subdomain IIA contains the protein’s main intrinsic fluorophore, a tryptophan residue (Trp-214), whose fluorescence is extremely sensitive to its microenvironment [6]. Hence, this Trp residue is often used to probe HSA’s structural changes upon ligand binding using various fluorescence spectroscopy techniques [7].

The cell-permeable nucleoside, 5-aminoimidazole-4-carboxamide ribonucleoside (AICAR) and its structural derivatives have recently received a great deal of attention for their broad-spectrum anti-cancer activity profiles [8]. AICAR activates 5′-AMP-activated protein kinase (AMPK), a master cellular energy sensor, coordinating metabolic pathways that balance nutrient supply with energy demand [9]. Recent studies have shown that AICAR’s cytotoxic and proapoptotic effects against various in vitro models of human cancer is in-part mediated through the AMPK signaling network [9,10]. AICAR has very few cytotoxic effects on most normal counterpart cells, including human fibroblast cells, thus mostly acting as a cancer-specific cytotoxic agent [9,10]. While there are numerous literature reports on AICAR’s bioactivity, the characterization of its drug-like properties and pharmacokinetics remains incomplete and thus warrants further study. Since the interaction of anti-cancer agents with plasma proteins, including HSA, significantly influences their pharmacokinetic properties, we aim to characterize the structural and biophysical basis for AICAR–HSA binding using experimental and computational techniques. The detailed analysis of AICAR–HSA binding interactions can provide valuable insights about AICAR’s in vivo pharmacokinetic properties, as well as HSA’s potential as a drug delivery system in future studies [11].

## 2. Results and Discussion

### 2.1. Protein Fluorescence Quenching of HSA by AICAR

**Figure 1:**
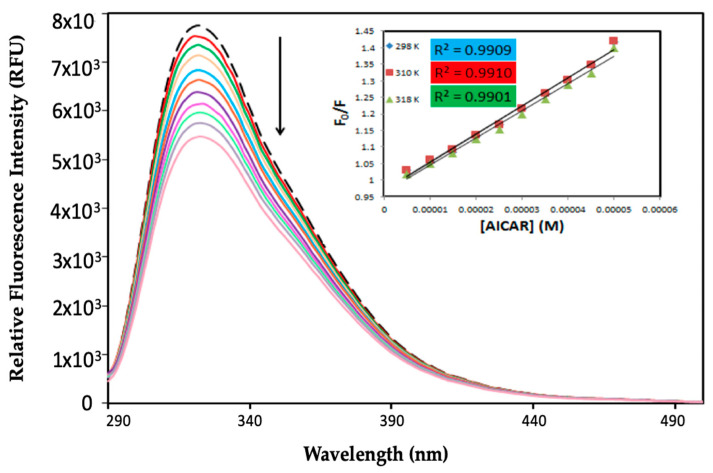
Fluorescence quenching spectra of 5-aminoimidazole-4-carboxamide ribonucleoside–human serum albumin (AICAR–HSA) complexes. Fixed [HSA] = 5 μM, increasing concentration of [AICAR] = 5–50 μM, pH = 7.4, T = 298 K, in 10x-PBS buffer.

Fluorescence spectroscopy has been widely used to investigate the intermolecular interactions between small ligands and proteins, leading to various ligand-induced changes in protein biophysical properties, including fluorescence quenching, binding affinity, energy transfer, and conformational dynamics [12]. Herein, fluorescence quenching refers to any process (i.e., binding of a ligand) that decreases the protein fluorescence intensity because of the changes in the fluorophore’s microenvironment. Protein fluorescence quenching, as well as a shift in the maximum emission wavelength (*λ*_em_) upon ligand–protein complex formation, can help profile the specific protein-binding sites and binding energies. The intrinsic fluorescence spectra of HSA in the absence and presence of AICAR in increasing concentrations are shown in Figure 1. The fluorescence emission spectrum of HSA displays *λ*_em_ of 322 nm at an excitation wavelength (*λ*_ex_) of 280 nm.

AICAR caused progressive fluorescence quenching of HSA without changing *λ*_em_ or the shape of the peaks. There was an approximately 30% decrease in HSA fluorescence intensity at the highest AICAR concentration (50 μM). This suggests that AICAR specifically interacts with HSA and quenches its intrinsic fluorescence in a concentration-dependent manner.

### 2.2. Fluorescence Quenching Mechanism

Fluorescence quenching of a protein in the presence of a ligand (i.e., a quencher) can occur through either a dynamic or static mechanism [12]. Dynamic fluorescence quenching stems primarily from transient intermolecular collisions between the protein and the quencher. Thus, the rate constant for quenching (*K_q_*) increases with increasing collision frequency in a temperature-dependent manner. Furthermore, dynamic fluorescence quenching does not require actual ligand binding, leading to no change in the protein’s conformational structure or function. In contrast, static fluorescence quenching primarily results from the formation of a stable bound protein–quencher complex. Thus, the quenching constant (*K_q_*) increases with decreasing diffusion frequency, representing an inverse dependence upon temperature [13,14].

Dynamic and static quenching can be distinguished by both biomolecular quenching rate constants (*K_q_*) and their contrasting dependence upon temperature. The maximum *K_q_* value for a collision-controlled dynamic quenching mechanism is typically 7.4 × 10^9^ L∙mol^−1^∙s^−1^ (298 K), while a higher *K_q_* value indicates a binding-controlled static mechanism [15].

The mathematical relationship between protein fluorescence quenching as a function of quencher concentration is described by the Stern–Volmer equation. This model is often used to predict the mechanism of fluorescence quenching [16]:

*F*_0_/*F* = 1 + *K_q_τ*_0_*[Q]* = 1 + *K_SV_[Q]* (1)

The quenching rate constant is calculated using the following equation:

*K_q_* = *K_SV_*/*τ*_0_ (2)

Here, *F*_0_ and *F* represent the equilibrium fluorescence intensities in the absence and presence of quencher, respectively. *K_SV_* is the Stern–Volmer constant, *[Q]* is the quencher concentration, *K_q_* is the biomolecular quenching rate constant, and *τ*_0_ is the average lifetime of the protein in the absence of any quencher.

The relative fluorescence intensity data (*F*_0_/*F*) as a function of quencher concentration was obtained at three different temperatures (298 K, 310 K, and 318 K). The values of *K_sv_* are determined by the slope of the linear function of *F*_0_/*F* vs. the quencher concentration *[Q]*. To calculate the *K_q_*, the average literature value of the fluorescence lifetime for HSA in the absence of any quenchers of (*τ*_0_ = 10^−8^ s) was used [17].

The calculated values of *K_SV_* and *K_q_* for the interaction of AICAR with HSA at three different temperatures are listed in Table 1. The values of both *K_SV_* and *K_q_* decrease with increasing temperature, suggesting a static fluorescence quenching mechanism for AICAR–HSA complex formation. The values of *K_q_* were approximately two orders of magnitude larger than the limiting bimolecular diffusion rate constant (K_diff_ = 7.4 × 10^10^ L∙mol^−1^∙s^−1^ at 298 K), further validating a static quenching mechanism that involves the formation of a ground state AICAR–HSA complex at equilibrium.

### 2.3. Thermodynamic Binding Constants and Interaction Sites between HSA and AICAR

#### 2.3.1. Thermodynamic Binding Parameters

The thermodynamic equilibrium-binding constant (*K_b_*) for the AICAR–HSA interaction and the number of AICAR binding sites (*n*) on HSA were calculated from a double logarithmic plot of *log* (*F*_0_ − *F*)/*F* versus *log[Q]*. This method employs the following equation [18]:

*log* (*F*_0_ − *F*)/*F* = *logK_b_* + *nlog[AICAR]* (3)

**Figure 2:**
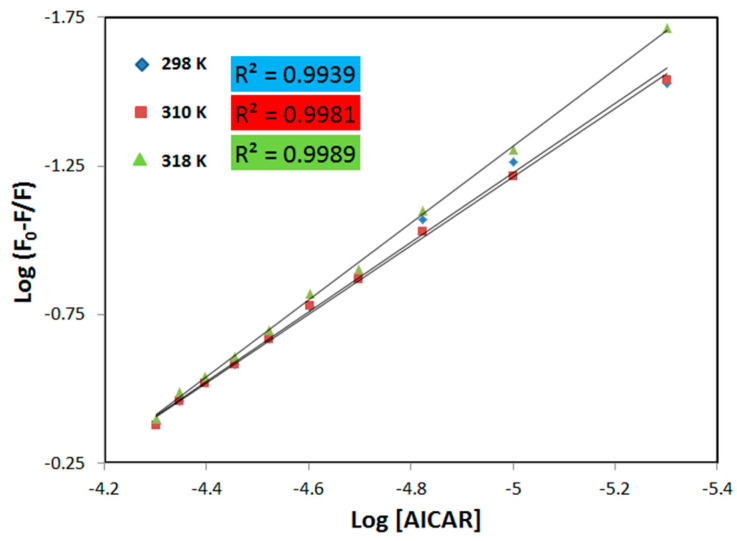
The plot of *log*[(*F*_0_ − *F*)/*F*] versus *log[Q]* for HSA in the presence of AICAR at three different temperatures. Fixed [HSA] = 5 μM, increasing concentration of [AICAR] = 5–50 μM, pH = 7.4, in 10x-PBS buffer.

The values of *n* and *K_b_* were obtained from the slope, and the y-intercept of these plots (Figure 2), respectively and are listed in Table 2. The *K_b_* values for the association of AICAR and HSA are in the range of 10^3^–10^4^ M^−1^, showing a moderate affinity between these two substances [18] (Table 2). The increase in *K_b_* values with increasing temperature indicates that the protein can better accommodate the ligand at 318 K than at 298 K, which may be due to HSA’s binding pocket being more accessible to AICAR at the higher temperature. The change in Gibbs free energy (*∆G*) of the AICAR–HSA complex formation under experimental conditions (pH = 7.4; T = 298, 310 and 318 K) was also determined using the following equation [18]:

*∆G* = −*RTlnK_b_* (4)

Here, *K_b_* is the binding constant calculated by Equation (3), *T* is temperature (K), and *R* is the universal gas constant (8.314 JK^−1^mol^−1^). As shown in Table 2, the negative *∆G* indicates that the formation of the AICAR–HSA complex is exergonic and thus thermodynamically favorable.

#### 2.3.2. Site-Specific Ligand Competitive Displacement Experiments

HSA is a major serum transporter protein for a variety of ligands, such as fatty acids, steroids, and drugs, because of its multiple hydrophobic ligand-binding pockets. It has three reversible ligand-binding sites with fairly distinct structural requirements for the ligands [19,20]. Sudlow et al. [6,19] have suggested two principal regions of reversible ligand binding sites on the HSA protein (sites I and II). Site I, located within subdomain IIA, has a pocket that preferentially binds to drugs with bulky heterocyclic scaffolds, such as warfarin, bilirubin, phenylbutazone, etc. Site II, located within subdomain IIIA, has a binding pocket that preferentially binds to smaller drugs with aromatic rings such as ibuprofen, flufenamic acid, tryptophan, etc., [20,21]. Digitoxin, a lipid soluble glycoside, is believed to bind to site III, a binding groove located in subdomain IB of HSA [22,23].

**Figure 3:**
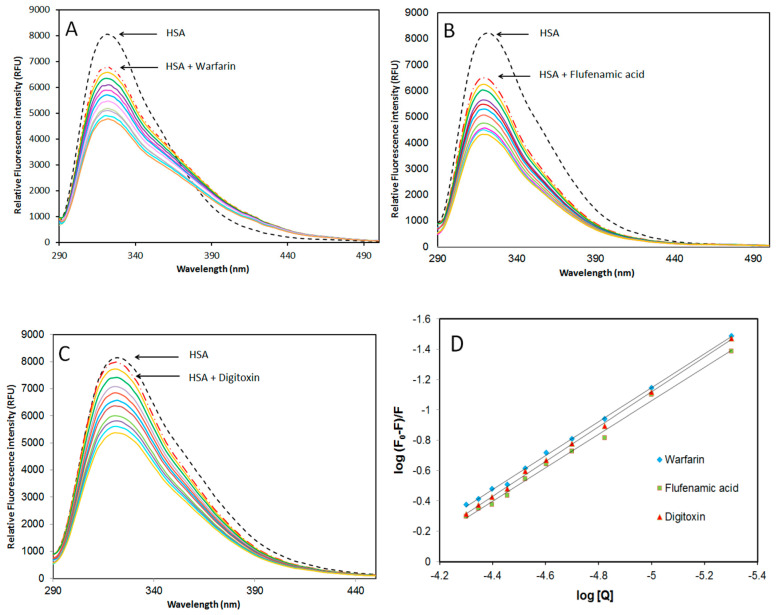
Effect of site marker probes (**A**) warfarin; (**B**) flufenamic acid; (**C**) digitoxin; and (**D**) double logarithmic plots of *log* (*F*_0_ − *F*)/*F* versus *log[Q]* for HSA-AICAR complexes. AICAR (5–50 μM), fixed HSA and probe concentrations at 5 μM, pH 7.4, 10x-PBS buffer at 298 K.

To determine AICAR’s preferred binding site on HSA, site marker competitive displacement experiments were performed with warfarin (specific for site I), flufenamic acid (specific for site II), and digitoxin (specific for site III) as control ligands at 298 K. For this analysis, varying amounts of AICAR (5–50 μM), were added to a solution containing fixed amounts of HSA (5 μM) and site probe (5 μM), and the relative fluorescence units (RFU) at the maximum emission wavelength (*λ*_em_ = 322 nm) was measured upon excitation at (*λ*_ex_ = 280 nm, Figure 3).

The values of the apparent binding constants in the presence of site markers were evaluated using Equation (3), and the results are listed in Table 3. As evident from these results, AICAR–HSA binding in the presence of warfarin and flufenamic acid was reduced by 32.5% and 28.9% respectively, when compared to AICAR–HSA without any site marker. This suggests that flufenamic acid and warfarin compete and displace AICAR from binding sites I and II on HSA. Therefore, AICAR may bind at both site I in subdomain IIA and site II in subdomain IIIA of HSA. On the other hand, in the presence of digitoxin, the decrease in K_b_ value was negligible, suggesting that AICAR does not bind in site III and its binding is independent of digitoxin binding [22].

### 2.4. Characterization of Conformational Change in HSA upon AICAR Binding

#### 2.4.1. Synchronous Fluorescence Spectroscopy

Synchronous fluorescence spectroscopy is a sensitive method used to probe changes in the binding pocket conformation and polarity upon ligand binding to a protein [24]. Often, ligand-induced protein conformational changes lead to binding pocket realignment which can affect the microenvironment of nearby HSA’s fluorophores, Trp (214) and Tyr (150, 411, 452) residues.

Here, the monochromators were set for synchronous scans at fixed wavelength differences (Δ*λ*) between the excitation wavelengths (*λ*_ex_) and the measured emission wavelengths (*λ*_em_) *for AICAR–HSA* complexes. Upon AICAR binding, conformational rearrangements leading to changes in the microenvironment of HSA fluorophores, Tyr and Trp residues, can be measured when the Δ*λ* is set at 15 nm and 60 nm respectively. The synchronous fluorescence data at Δ*λ* = 15 nm and Δ*λ* = 60 nm are summarized in Table 4.

**Figure 4:**
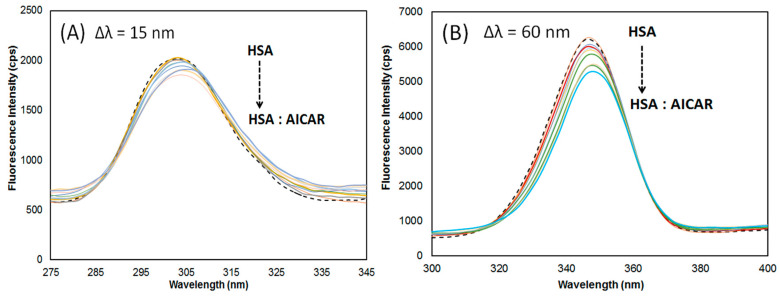
Synchronous fluorescence spectra of AICAR–HSA complex with varying AICAR concentration (5–50 μM) and fixed HSA concentration (5 μM); (**A**) Δ*λ* = 15 nm, and (**B**) Δ*λ* = 60 nm, at pH 7.4 and 298 K.

As seen in Figure 4, at ∆*λ* = 60 nm, the synchronous fluorescence intensity of HSA decreased with increasing AICAR concentration by ~6%, consistent with conventional fluorescence quenching data observed earlier. Furthermore, a slight but persistent red shift of 2 nm in maximum *λ*_em_ was observed. When probing for Tyr at ∆*λ* = 15 nm, although the fluorescence intensity of HSA decreased with increasing AICAR concentration by ~9%, there was no obvious blueshift or redshift in the maximum *λ*_em_. This suggests that the AICAR binding mostly affects the binding pocket conformation near Trp-214 in site I and thus its microenvironment polarity, with negligible effects on conformation near the Tyr residues (411, 452) in site II.

#### 2.4.2. Three-Dimensional Fluorescence Spectroscopy

**Figure 5:**
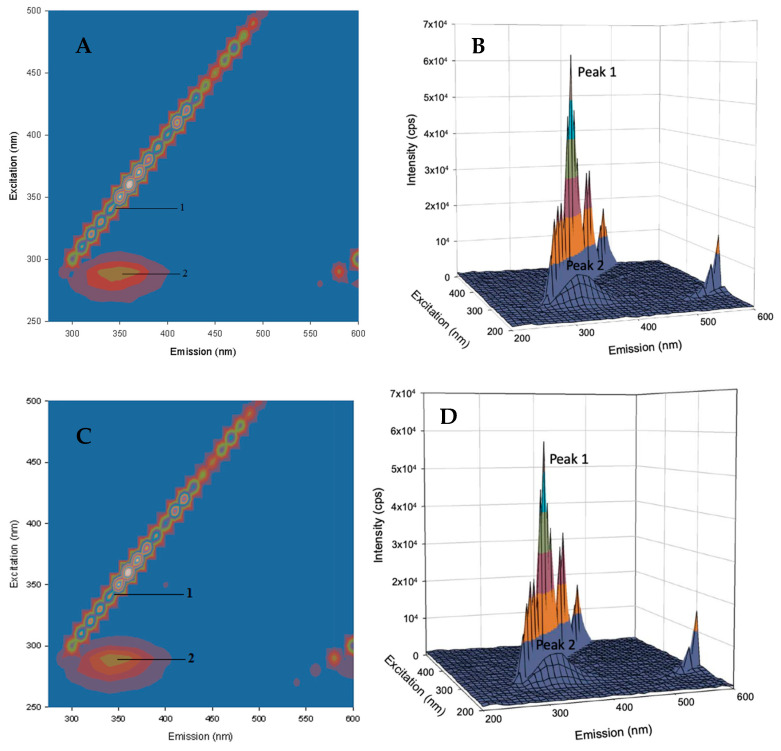
The 3D fluorescence contour map of free HSA (5 μM) (**A**) and the bound AICAR–HSA complex after addition of AICAR (5 μM) (**C**). The 3D fluorescence projections of free HSA (5 μM) (**B**) and bound AICAR–HSA complex after addition of AICAR (10 μM) (**D**).

Three-dimensional fluorescence spectroscopy is an effective method for studying protein structural dynamics upon ligand binding [25,26]. In this experiment, the three axes of the fluorescence spectra are the excitation wavelength (*λ*_ex_), the maximum emission wavelength (*λ*_em_), and the fluorescence intensity. Figure 5 presents the 3D fluorescence spectra and the corresponding contour maps for free HSA and AICAR–HSA-bound complexes, the detailed descriptions of peaks I and II are outlined below:

Peak I (*λ*_ex_ = 360 nm, *λ*_em_ = 360 nm) is the *Rayleigh* scattering peak, which reflects changes in the overall protein surface area as a result of ligand binding. For complexes of AICAR–HSA (1/1 and 2/1 ratios), the intensity of this peak decreased (~7%) and (~13%) respectively with no shift in maximum *λ*_em_. This suggests a moderate decrease in the total protein surface area upon AICAR binding in a concentration-dependent manner.

Peak II (*λ*_ex_ = 280 nm, *λ*_em_ = 340 nm) is due to intrinsic fluorescence of HSA’s main fluorophore, Trp214 residue and reflects changes in its microenvironment polarity as a result of ligand binding. For the 1/1 AICAR–HSA complex, we observed a moderate decrease in fluorescence intensity (~8%) with no red or blue shift in the maximum *λ*_em_. However, for the 2/1 AICAR–HSA complex, we observed a slightly more decrease in fluorescence intensity (9%) and but a significant 10 nm red shift in the maximum *λ*_em_.

Overall, in the presence of AICAR, the fluorescence intensities of peaks I and II decreased in a concentration-dependent manner, in agreement with the steady-state and synchronous fluorescence quenching observed in previous experiments. Furthermore, a 10 nm red shift observed in the maximum *λ*_em_ for peak II (AICAR–HSA, 2/1) indicates that ligand binding flexed the HSA’s binding site I conformation and thus altered the microenvironment polarity around HSA’s main fluorophore, Trp214 (Table 5). This observation further corroborated the synchronous fluorescence data.

#### 2.4.3. UV-VIS Absorption Spectroscopy

**Figure 6:**
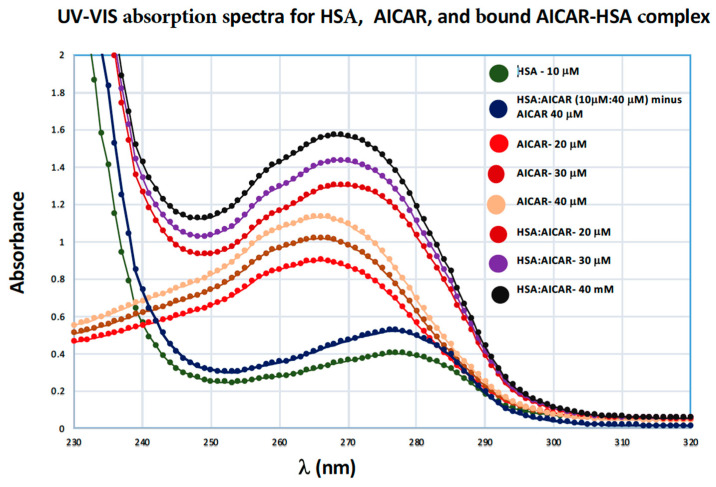
UV-VIS absorption spectra of free HSA (10 μM), AICAR–HSA (40:10 μM) subtracted from AICAR 40 μM, free AICAR (20, 30, 40 μM), AICAR–HSA complexes (20:10, 30:10, 40:10 μM), pH 7.4, 10x-PBS buffer at 298 K.

UV-VIS absorption spectroscopy is a basic spectrometric method used to investigate structural changes that accompany the formation of bound ligand–protein complexes [27,28]. The aromatic amino acids in proteins, tryptophan and tyrosine, strongly contribute to UV absorption of proteins at 280 nm and 274 nm respectively. The UV-Vis absorption spectum for the free AICAR ligand, free HSA, and bound HSA/AICAR complexes is shown in Figure 6. Here we see that HSA in its AICAR-bound form (at all three different AICAR concentrations) displays a distinct *λ*_max_ value at 269 nm, which represents an 8-nm blue shift from the *λ*_max_ value of free HSA and a 3-nm red shift from *λ*_max_ value for free AICAR (Figure 6). We also see a concentration-dependent increase in HSA’s peak intensity for the bound AICAR–HSA complexes. This data reaffirms that HSA and AICAR form a new molecular entity that has its own characteristic UV absorption, one that is distinct from the free AICAR and the free HSA. The data also corroborates our original spectral analysis and confirms the existence of a true bound AICAR–HSA complex.

### 2.5. Fluorescence Energy Transfer between HSA and AICAR–HSA

Fluorescence resonance energy transfer (FRET) is a prominent physical phenomenon used to investigate the molecular interactions of ligands with fluorescent proteins [29,30]. In FRET, the non-radiative resonance energy is transferred over greater than interatomic distances without conversion to thermal energy, and without any molecular collision. FRET leads to a reduction in the donor’s (protein fluorophore) fluorescence intensity and excited state lifetime, and an increase in the acceptor’s (ligand) emission intensity [31].

**Figure 7:**
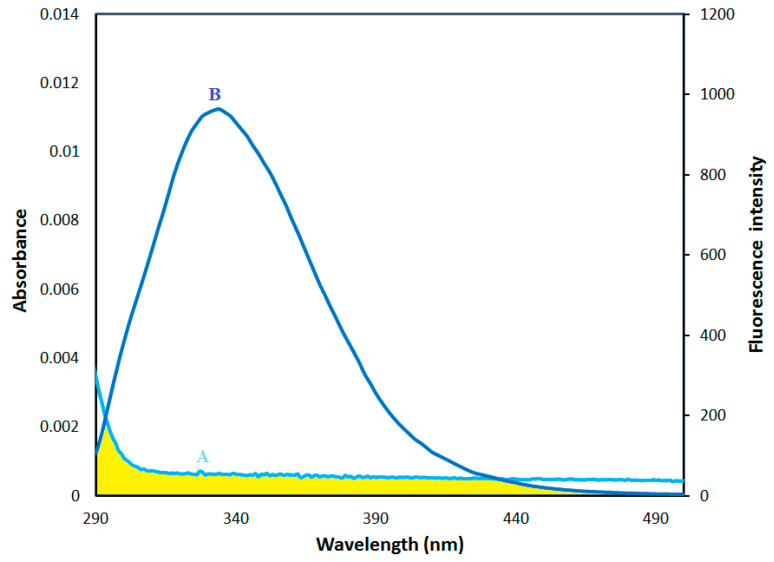
The overlap of (A) UV absorption spectra of AICAR (5 μM) with (B) fluorescence spectra of HSA (5 μM), (*λ*_ex_ = 280 nm).

For an efficient fluorescence energy exchange between donor and acceptor, three conditions must be met. First, the donor must have inherent fluorescence; second, there must be a fair amount of overlap between the donor’s fluorescence emission spectrum and the acceptor’s absorption or excitation spectrum, and finally, the donor and acceptor molecules must be in close proximity to one another (~1–10 nm) [31]. Thus, FRET is an effective tool for evaluating the distance between the protein donor and the ligand in the bound ligand–protein complexes [31]. The overlap of the UV absorption spectrum of AICAR with the fluorescence emission spectrum of HSA is shown in Figure 7.

The energy transfer efficiency and the distance between the donor (Trp214 of HSA) and the acceptor (AICAR) can be calculated using the theoretical model of Förster non-radiative energy transfer, Equation (5) [30,31]:

(5) $$E\;=\;\frac{R_{0}^{6}}{R_{0}^{6}+r^{6}}\;=\;\frac{F_{0}-F}{F_{0}}$$

Here, *F* and *F*_0_ are the fluorescence intensities of HSA in the presence and absence of AICAR respectively; r is the distance between the donor and the acceptor, *R*_0_ is the critical energy transfer distance when the transfer efficiency is 50%; and E is the specific energy transfer efficiency (*E*). The value of *E* was calculated using fluorescence intensities of HSA (5 μM) with and without AICAR (5 μM) at 298 K using (*λ*_ex_ = 280). The value of *R*_0_ was evaluated using Equation (6):

*R*_0_ = 0.211 × (*K*^2^ × *n*^−4^ Φ_D_*J*(*λ*))^1/6^ (6)

Here, *K*^2^ represents the spatial orientation factor related to the relative dipole directions of the donor/acceptor. *n* is the average refractive index of the medium (10X-PBS) in the wavelength range where there is a notable fluorescence-absorption spectral overlap (295–440 nm). Φ_D_ is the fluorescence quantum yield of the donor HSA.

J is the spectral overlap integral of the fluorescence-absorbance spectra for the donor–acceptor pair (Figure 7). *λ* is the maximum emission wavelength of HSA (*λ*_em_ = 302 nm) as a fluorescent donor. The value of *J* can be calculated using Equation (7):

(7) $$J\;=\;\frac{\Sigma F\left(\lambda \right)\varepsilon \left(\lambda \right)\lambda^{4}∆\lambda }{\Sigma F\left(\lambda \right)∆\lambda }$$

Here, *F*(*λ*) represents the fluorescence intensity of the fluorescent donor at emission wavelength *λ*, and ε(*λ*) represents the molar absorption coefficient of the acceptor at the absorption wavelength *λ*_max_. The literature values of 2/3, 0.15, and 1.336 were used for K^2^, Φ_D_ (HSA), and n (10x-PBS) respectively [31,32].

Using Equations (5)–(7), the values of *J*, *R*_0_, *E*, and *r* were calculated to be *J* = 9.42 × 10^12^ cm^3^∙L/mol, *R*_0_ = 1.72 nm, *E* = 0.06, and *r* = 2.72 nm, respectively, at 298 K.

The calculated value of *R*_0_ = 1.72 nm and *r* = 2.72 nm are within the range for FRET distal requirements, indicating an efficient intermolecular energy transfer and formation of a stable AICAR–HSA complex. This also further corroborates the previously stated static mechanism for fluorescence quenching that occurred in AICAR–HSA complexes.

### 2.6. Molecular Docking Studies for the Interaction AICAR–HSA

**Figure 8:**
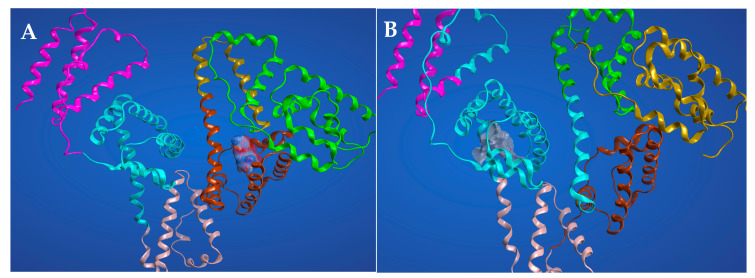
The best-scoring ribbon renditions for the AICAR–HSA complexes in Sudlow’s site I (**A**) and in Sudlow’s site II (**B**).

Competitive displacement studies suggest that AICAR has binding affinity for both HSA site I (warfarin site) and site II (flufenamic acid site). Thus, we performed the molecular docking experiments to probe for AICAR’s ability to dock into both sites I and II. The computational docking experiments were performed with 3-D x-ray structural models of three protein complexes, HSA complexed with heme (PDB-ID 1N5U, 1.90 Å), warfarin (PDB-ID 2BXD, 3.05 Å), and ibuprofen (PDB-ID 2BXG, 2.70 Å), using the molecular modeling system, *MOE-CCG* and *Autodock-vina* [33,34]. The ribbon renditions of the lowest energy AICAR–HSA complexes, representing the most stable binding modes (for sites I and II) are shown in Figure 8. The results predict that hydrogen bonding is the main non-covalent interaction that stabilizes the highest-scoring docking conformation of AICAR–HSA complexes at either binding sites. The predicted binding affinity scores for the bound complexes are −6.61 kcal/mol and −6.52 kcal/mol for site I and II respectively, suggesting that AICAR has a slightly higher affinity for site I than II, which corroborates the experimental data on site-specific competition assays presented earlier.

For AICAR binding at HSA site I, extensive H-bonds are donated by Glu-292 and Lys-195 residues to three ribose oxygen atoms at C1, C2, and C5. The most energetically favorable interaction is an H-bond from the amide hydrogens of 4-carboxamide to carbonyl oxygen on the side chain of Glu-153 (Figure 8). In addition, side chain carbonyl oxygens within Glu-153 and Glu292 serve as H-bond acceptors from the amide and amine hydrogens on AICAR’s 4-carboxamide and 5-aminoimidazole groups respectively. These interactions are predicted to occur at distances required for effective H-bonding ranging from 2.78 Å to 3.43 Å. For AICAR binding at HSA site II, the side chain carbonyl oxygen of Glu354 serves as an H-bond acceptor from the ribose hydroxyl group at C3 position (distance of 2.65 Å). In addition, the Lys-351 forms a notable hydrophobic aromatic π-stacking interaction with the imidazole ring of AICAR (distance 4.32 Å).

Overall, the molecular docking studies provide a useful framework for visualizing at the atomic level the specific ligand–protein interactions and the quantitative analysis of binding affinity scores, which are in good agreements with the site marker competition data presented earlier. The visualized output and molecular details for all docking experiments, including specific binding site interactions are included in the supplementary materials.

## 3. Materials and Methods

### 3.1. Materials

HSA, Uniprot ID: P02768 (99.0% purity by electrophoresis) and AICAR, Drug Bank ID: DB01700 (98.0% purity by HPLC) were purchased from Sigma (St. Louis, MO, USA) and Chem Cruz (Dallas, TX, USA), respectively. Warfarin, flufenamic acid, and digitoxin were purchased from Sigma-Aldrich, Alfa Aesar (Tewksbury, MA, USA) and TCI America (Portland, OR, USA), respectively. All other reagents were of analytical grade and used without further purification. Millipore deionized water was used in all experiments. Sample masses were accurately measured on a Mettler Toledo analytical balance (Columbus, OH, USA) with 0.1 mg precision, and all pH measurements were made using an *AB-15 Accumet Basic* pH meter (Waltham, MA, USA).

#### Preparation of Stock Solutions

The stock solutions of HSA (100 microM, M.W. 65.7 kDa), AICAR (1 mM, M.W. 258.23 g/mol), warfarin (1 mM, M.W. 308.33), flufenamic acid (1 mM, M.W. 281.23 g/mol), and digitoxin (1 mM, M.W. 764.94 g/mol) were prepared in 10x-PBS (137 mM NaCl, 10 mM phosphate, 2.7 mM KCl) at pH 7.4.

### 3.2. Major Equipment

#### UV-VIS Absorption Spectroscopy

The UV-Vis absorption spectra were recorded at room temperature on a double-beam *Lambda 25* spectrometer (Perkin Elmer, Waltham, MA, USA), equipped with 1.0 cm quartz cells. The UV measurements of HSA in the presence and absence of AICAR were made in the 200–400 nm range. HSA concentration was fixed at 5 μM while AICAR concentration was varied from 5 μM to 50 μM in the 10x-PBS buffer at pH-7.4.

### 3.3. Fluorescence Measurements

#### 3.3.1. Fluorescence Spectroscopy Experiments

Fluorescence measurements (counts per second, CPS) were carried out using a *SpectroMax M2/M2e* spectrometer (Molecular Devices, San Jose, CA, USA) from Molecular Devices equipped with *Cellstar 96* well plates (black with clear flat bottom, non-treated, no lid). The concentration of HSA was fixed at 5 μM, and the AICAR concentration was varied from 5 to 50 μM for the steady-state fluorescence experiments. Fluorescence spectra were recorded at 25 °C (298 K), 37 °C (310 K), and 45 °C (318 K) in the 290–500 nm range upon excitation at a wavelength of 280 nm [33]. All samples were prepared in 0.1 M phosphate-buffered saline (10x-PBS) at pH 7.4 and were incubated at each temperature for 30 min. Each experimental measurement was performed in triplicate to ensure reproducibility. Under identical conditions, there was no detectable fluorescence emission for AICAR in the concentration range of 5 to 50 μM.

#### 3.3.2. Synchronous Fluorescence Spectroscopy

The *Fluorolog* spectrometer (HORIBA, Ann Arbor, MI, USA) equipped with 1.0 cm quartz cells was used for measuring RFU for the synchronous and 3D-fluorescence experiments. Here, the monochrometers were set for the simultaneous scanning of excitation and emission at a fixed wavelength difference (∆*λ* = *λ*_em_ − *λ*_ex_). The excitation and emission slit widths were set at 1 nm. The scan speed was set at 240 nm/min. In each assay, HSA (5 μM) was titrated with AICAR (at concentrations from 5 to 50 μM). The spectral behavior of tyrosine and tryptophan residues of HSA was observed at Δ*λ* = 15 nm and Δ*λ* = 60 nm, respectively, at 25 °C.

#### 3.3.3. Site Marker Competitive Fluorescence Experiments

The competitive binding site studies were performed using three different site probes, namely warfarin, flufenamic acid, and digitoxin for sites I, II, and III, respectively, by keeping the concentration of HSA and the probe constant (5 μM each). The AICAR solution was varied from 5 to 50 μM with 30 min incubation periods at room temperature for each probe in PBS at pH 7.4.

#### 3.3.4. 3D Fluorescence Spectra

3D fluorescence spectra were recorded by scanning excitation wavelength in the 200–500 nm range and emission wavelength from 200–600 nm at 10 nm intervals for 5 μM of protein solution with and without 5 μM and 10 μM AICAR. The scanning parameters were the same as in the fluorescence quenching experiments.

### 3.4. Characterization of AICAR–HSA Binding Interactions by Molecular Docking

#### 3.4.1. Protein Preparation

The 3-D modeled structures of each HSA protein molecule complexed with heme (1N5U), warfarin (2BXD), and ibuprofen (2BXG) were downloaded in PDB format from the RCSB database. The PDB files were input into the Molecular Operating Environment (MOE) software (ACA-ANS, 2019.01) [33,34]. The *MOE-QuickPrep* tool was used to prepare the protein structure for the docking experiments. The semi-automated preparation steps included: Removing the unbound water molecules near the binding sites; adding all missing terminal residues; adding all of the hydrogens on polar atoms (oxygen and nitrogen), adjusting the protonation states of amino acids with ionizable side chains; and correcting the poorly resolved X-ray structural discrepancies and anomalies in side chain conformation; and energy minimization using MMFF94 to the RMSD gradient value of 0.001. The default physiological conditions (T = 300 K, pH = 7.0, salt-conc. = 0.1 M) were used for setting up the *Protonate 3D* within *QuickPrep*.

#### 3.4.2. Ligand Preparation

The ligand structures for AICAR (CID:17513), flufenamic acid (CID:3371), warfarin (CID:54678486) were downloaded in sdf format from the *PubChem* database. The structures in CID file formats were input into *Spartan ’18 parallel Suite* (Wavefunction, Inc., Irvine, CA, USA). The ligand structures were energy minimized to determine the lowest energy conformations in an aqueous environment with the density functional theory (*DFT*), method *Becke-3-Lee Yang Parr (B3LYP)*, and the standard 6-31G* basis set.

#### 3.4.3. Docking

Standard searching and scoring functions of MOE were used to predict the molecular depiction of the binding pocket, interacting residues, and the binding energies for the computationally docked ligand–protein models [35]. The binding sites for the ligands were first searched on each of the prospective protein structures, using *MOE’s* triangle matcher placement function. The London ΔG placement scoring function was then used to score and rank the binding sites on the basis of binding energy. Using *MOE’s* induced fit refinement function, the conformational ensemble for the ligand–protein bound complexes were sampled for refinement and the most favored bound conformation was selected when the RMSD gradient value fell below 0.01 Å. The maximum number of iterations for the minimization process was set at 500 kcal/mol. Then, the highest-affinity bound conformations generated by the refined induced fit protocol were rescored using the refined affinity ΔG docking scoring function in MOE.

## 4. Conclusions

In this study, we have systematically analyzed the structural and biophysical basis of molecular interactions of AICAR and HSA using multiple experimental and modeling methodologies. We primarily relied on the sensitivity of HSA’s main fluorophore, Trp-214, in our experimental characterization of ligand binding, steady-state fluorescence quenching, protein conformational change, and FRET validation. The steady-state fluorescence experiments indicate that the formation of AICAR–HSA leads to a significant decrease in the fluorescence of Trp-214 in a concentration-dependent manner. The analysis of the Stern–Volmer fluorescence parameters, K_SV_ and K_q_, reveal an inverse correlation with temperature, indicating the formation of stable AICAR–HSA complexes. Furthermore, fluorescence quenching occurred predominantly through a collision-independent static mechanism. The values of *K_q_* were approximately two orders of magnitude larger than the limiting bimolecular diffusion rate constant (K_diff_ = 7.4 × 10^10^ L∙mol^−1^∙S^−1^ at 298 K), further validating a static fluorescence quenching mechanism.

The characterization of thermodynamic binding parameters for the AICAR–HSA complex formation reveal a moderate association constant and change in Gibbs free energy (K_b_ = 4.18 × 10^−5^ M^−1^, ∆G = −26.4 kJ/mol at 298K). The negative ∆G indicates that the overall AICAR–HSA interactions were thermodynamically favorable and thus spontaneous. Using Förster’s theory, FRET parameters of *R*_0_ = 1.72 nm and r = 3.05 nm are within the range for FRET distal requirements, further validating the formation of stable AICAR–HSA complexes, robust intermolecular interactions, and an efficient energy transfer from Trp-214 to AICAR, causing fluorescence quenching.

Using ligands with known binding sites in competitive displacement experiments, including warfarin (site I, subdomain IIA), flufenamic acid (site II, subdomain IIIA), and digitoxin (site III, subdomain IB), we found that AICAR has dual binding affinity for HSA′s prominent binding sites I and II. The collective experimental data from synchronous fluorescence and 3D-fluorescence spectroscopy demonstrate that AICAR binding triggers a moderate conformational change in the microenvironment of the Trp214 fluorophore. This may explain why Trp-214′s characteristic fluorescence is more affected by AICAR binding in site I.

Molecular docking results suggest that AICAR interacts via several non-covalent hydrogen bonding interactions with Glu292, Glu153, Lys195, and Glu199 in Sudlow’s site I and with Glu354 and Lys351 in Sudlow’s site II. These results are in good agreement with prior data from ligand competition experiments. While AICAR does not share significant similarities with warfarin and flufenamic acid in terms of size, structure, or hydrophobicity, it binds to HSA’s site I and site II with comparable affinity respectively. This is not unprecedented, since prior studies have characterized HSA as a unique monomeric protein with remarkable allosteric properties and conformationally adaptable binding pockets (sites I and II) [35]. HSA offers various ways of accommodating structurally diverse compounds with broad fitting requirements in either site I and II [1].

A drug’s affinity to bind to human serum albumin (HSA) is an important consideration in drug development, because it generally influences drug absorption, distribution, and elimination, and therefore its bioavailability [35]. AICAR is a small bioactive molecule with promising therapeutic potential against human cancer. Herein, we have systematically characterized AICAR’s binding to HSA, using a variety of experimental, theoretical, and computational methods, including fluorescence, UV-vis spectroscopy, Stern–Volmer theory, and molecular docking. This study provides new biochemical and structural information on AICAR’s binding to HSA that is valuable for the evaluation of its therapeutic potential and for future rational drug design studies.

## Figures and Tables

**Table 1:** Quenching rate constants and Stern–Volmer constants for HSA–AICAR complex.

T (K)	Kq (M^−1^ s^−1^) × 10^11^	Ksv (M^−1^) × 10^3^	R^2^
298	8.539	8.539 ± 0.293	0.991
310	8.653	8.653 ± 0.337	0.988
318	8.223	8.223 ± 0.292	0.990

**Table 2:** Thermodynamic binding parameters of the AICAR–HSA complex.

T (K)	K_b_(M^−1^) × 10^3^	*n*	R^2^	ΔG (kJ·M^−1^)
298	38.301 ± 0.00146	1.161 ± 0.0356	0.9925	−26.15
310	48.031 ± 0.00120	1.181 ± 0.0170	0.9983	−27.78
318	139.064 ± 0.00119	1.292 ± 0.0161	0.9988	−31.31

*K_b_*, binding constant; R, linear correlation coefficient; *n*, the number of binding sites.

**Table 3:** Equilibrium binding constants in site-competitive displacement experiments at 298 K.

T (K)	System	K_b_(M^−1^) × 10^3^	*n*	R^2^	ΔG (kJ·M^−1^)
298	AICAR–HSA	38.30 ± 0.001	1.16 ± 0.04	0.9925	−26.15
298	AICAR–HSA–Warfarin	28.38 ± 0.001	1.12 ± 0.01	0.9987	−25.40
298	AICAR–HSA–Digitoxin	40.18 ± 0.001	1.14 ± 0.01	0.9988	−26.27
298	AICAR–HSA–Flufenamic Acid	24.55 ± 0.001	1.08 ± 0.03	0.9953	−25.04

*K_b_*, binding constant; R, linear correlation coefficient.

**Table 4:** The synchronous fluorescence data for HSA and AICAR–HSA (1/1 ratio) (**A**) Δ*λ* = 15 nm, and (**B**) Δ*λ* = 60 nm.

**(A)**	**Intensity (cps)** **± SD**	**Wavelength (nm)**
HSA	4303 ± 100	302
AICAR–HSA	3910 ± 55	302
% Change	−9.14	
Shift		0
**(B)**	**Intensity (cps)** **± SD**	**Wavelength (nm)**
HSA	12,166 ± 219	346
AICAR–HSA	11,430 ± 255	348
% Change	−6.05	
Shift		2

**Table 5:** 3D fluorescence spectroscopy data for HSA/AICAR in ratios of 1/1 and 1/2.

System	Peak Number	Peak Position *λ*_ex_/*λ*_em_ (nm/nm)	Intensity (cps) ± SD	% Relative Intensity Change	Shift *λ*_em_ (nm)
HSA	1	360/360	252,756 ± 23,400		
	2	280/340	9080 ± 537		
AICAR–HAS (1:1)	1	360/360	235,360 ± 17,600	−6.9	0
	2	280/340	8393 ± 575	−7.6	0
AICAR–HSA (2:1)	1	360/360	220,564 ± 9960	−12.7	0
	2	280/350	8293 ± 269	−8.7	10

## Supplementary Materials

The following are available online. Figure S1: The modular structural organization of HSA; Figure S2: 2D binding interaction map- AICAR in HSA site-II; Figure S3: 2D binding interaction map- AICAR in HSA site-I; Figure S4a: Electrostatic surface maps for bound AICAR in HSA Sudlow site-I & II; Figure S5a: HSA (PDB:ID 2BXD) bound AICAR Sudlow site-I; Figure S5b: HSA (PDB:ID 1N5U) bound AICAR Sudlow site-I; Figure S5c. Binding pocket VDW interaction Surface map bound HSA:AICAR; Figure S6a. HSA (PDB:ID 2BXG) bound AICAR Sudlow site-II; Figure S6b. HSA (PDB:ID 1N5U) bound AICAR Sudlow site-II; Figure S6c. Binding pocket VDW interaction Surface map, HSA (PDB:ID 1N5U) bound AICAR Sudlow site-II; Figure S7: Ribbon Rendition of AICAR bound to HSA Site-I (PDB ID: 1N5U); Figure S8: Ribbon Rendition of AICAR bound to HSA Site-II (PDB ID: 1N5U).
