# Supplementary material for: Binding Studies of AICAR and Human Serum Albumin by Spectroscopic, Theoretical, and Computational Methodologies
Source: Molecules. 2020 Nov 19;25(22):5410. doi: 10.3390/molecules25225410 (PMC7699360; doi:10.3390/molecules25225410)

Figure 1: The modular structural organization of HSA (PDB ID:2BXG)

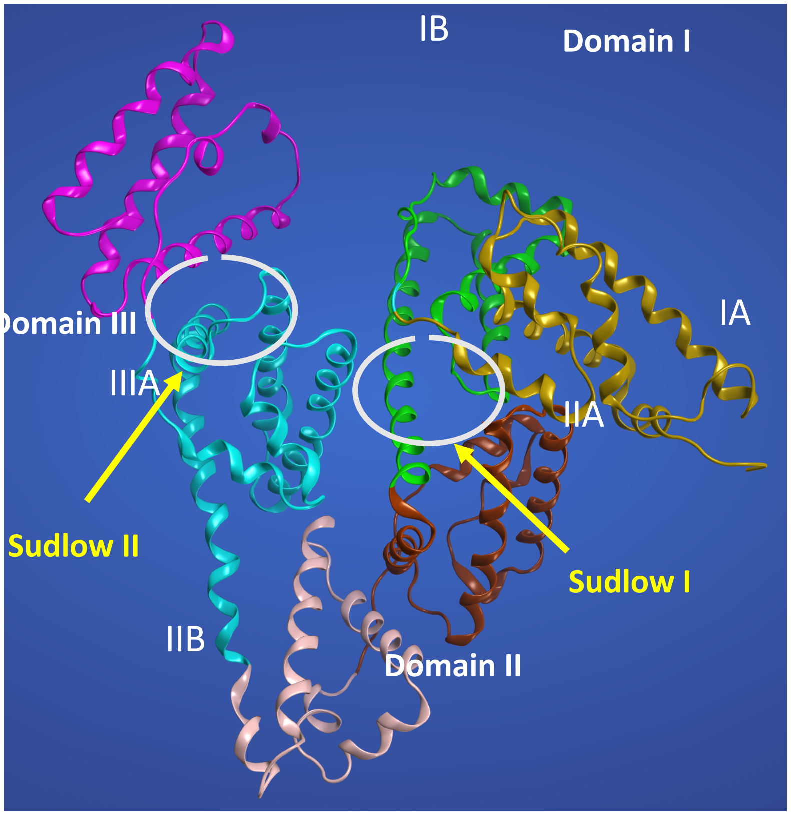

Figure 2: 2D binding interactions- AICAR in HSA site-II, (PDB ID:2BXG)

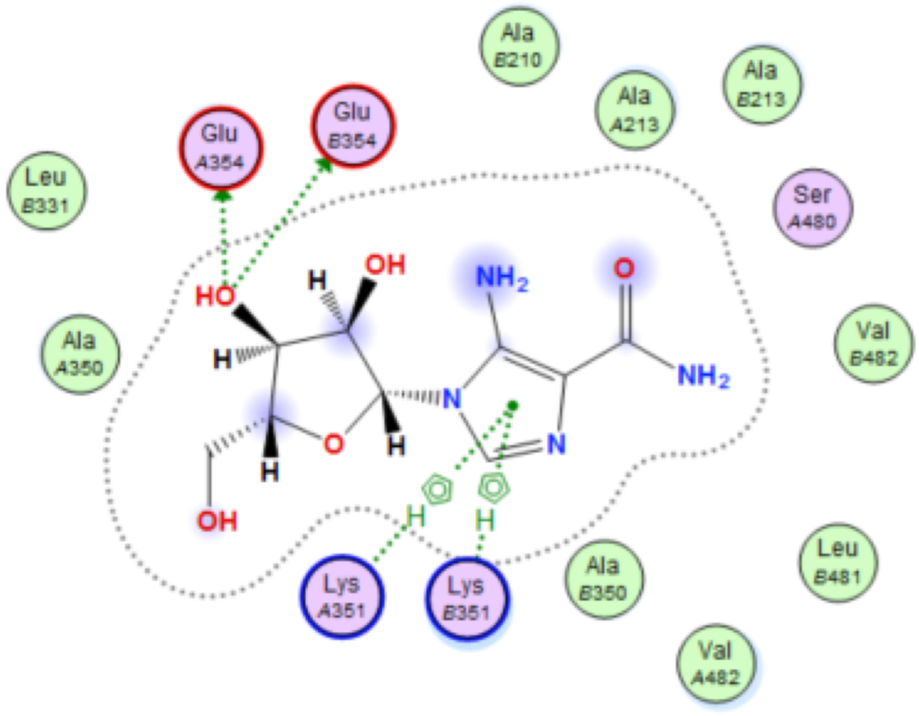

Figure 3: 2D binding interactions- AICAR in HSA site-I, (PDB ID:2BXD)

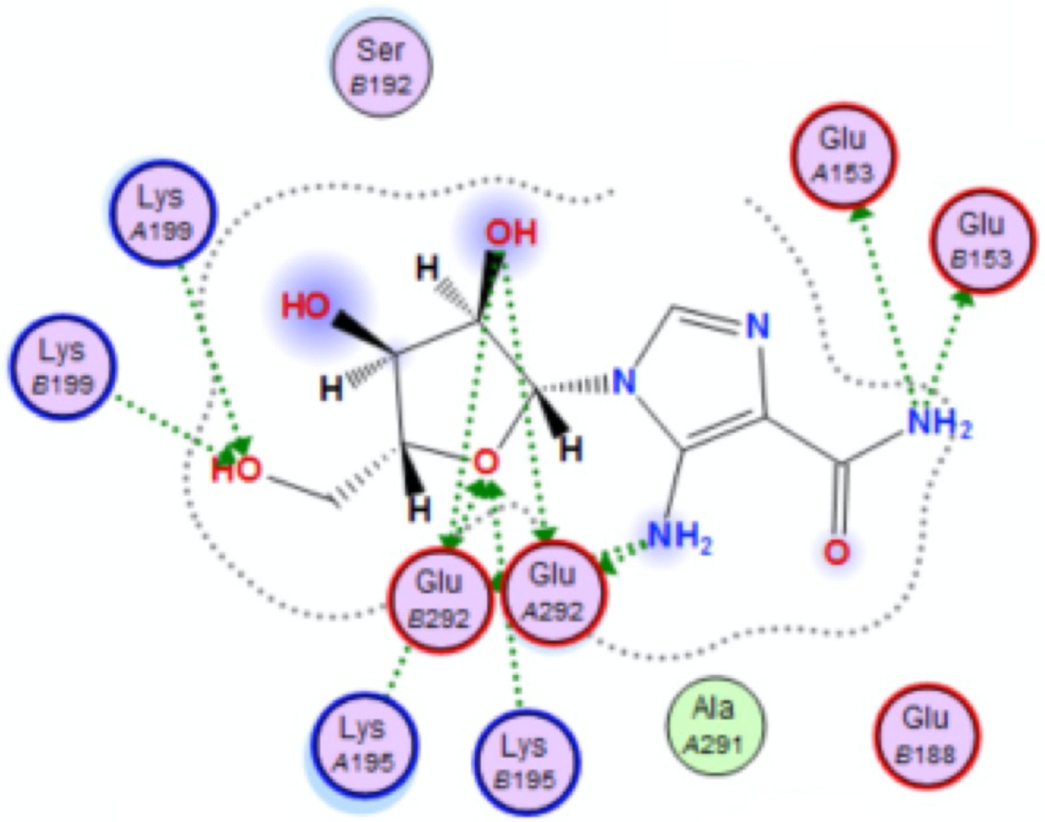

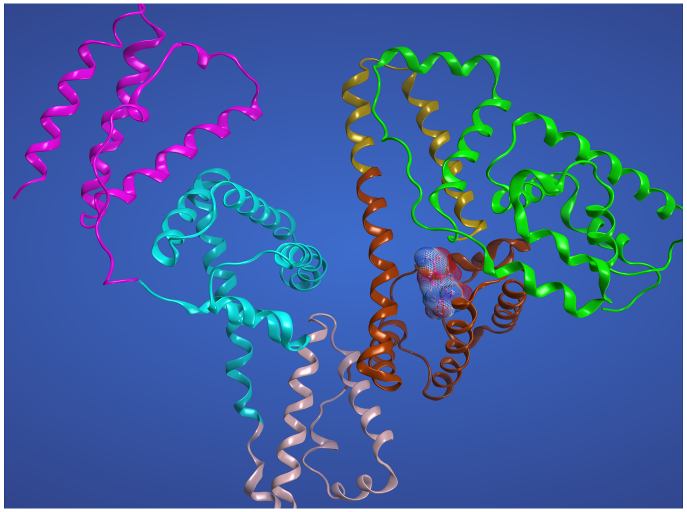

Figure 4a: Electrostatic surface map of bound AICAR in HSA (PDB:ID 1N5U) Sudlow site-I.

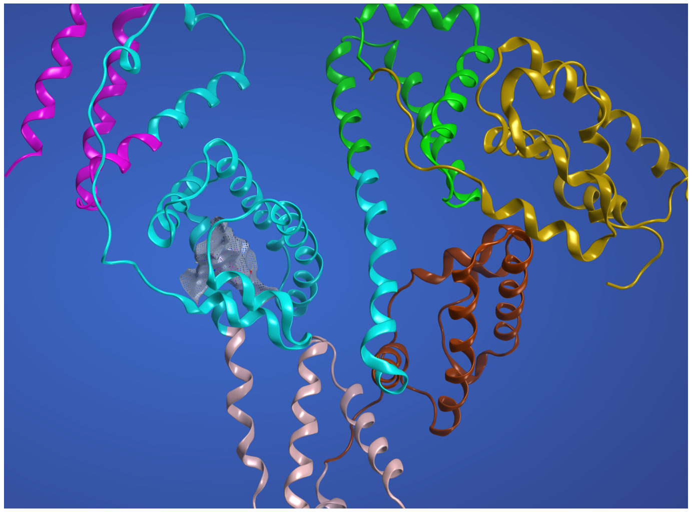

Figure 4b: Electrostatic surface map of bound AICAR in HSA (PDB:ID 1N5U) Sudlow site-II.

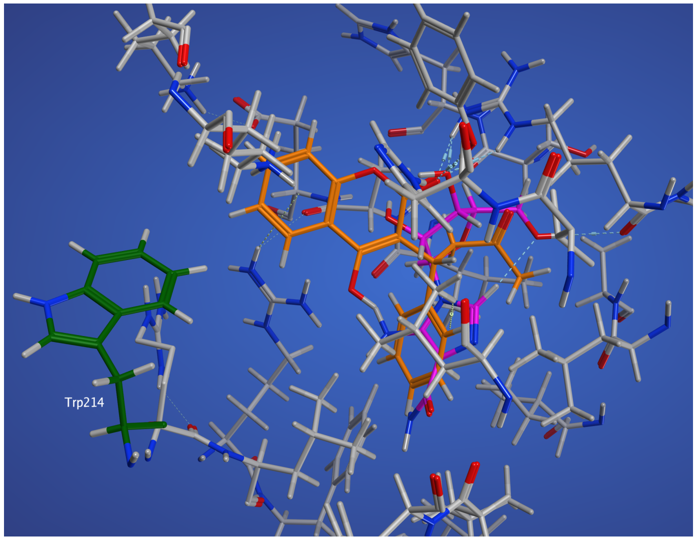


Figure 5a: HSA (PDB:ID 2BXD)

bound AICAR Sudlow site-I

AICAR (MAGENTA)

Warfarin (ORANGE)

Tryptophan 214 (GREEN)

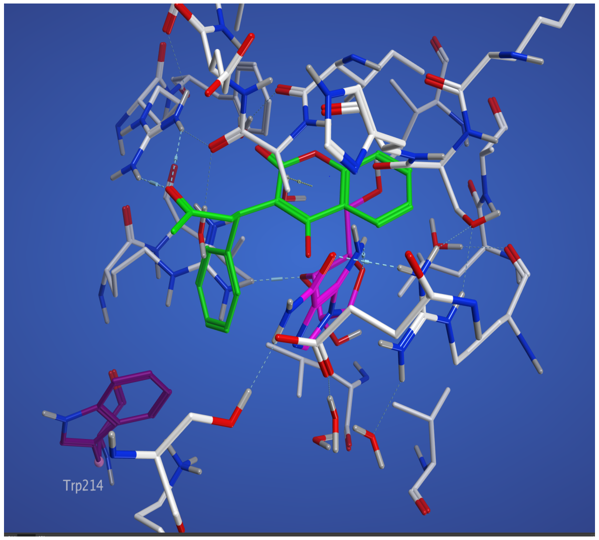


Figure 5b: HSA (PDB:ID 1N5U) bound

AICAR Sudlow site-I

AICAR (MAGENTA)

Warfarin (Green)

Tryptophan 214 (purple)

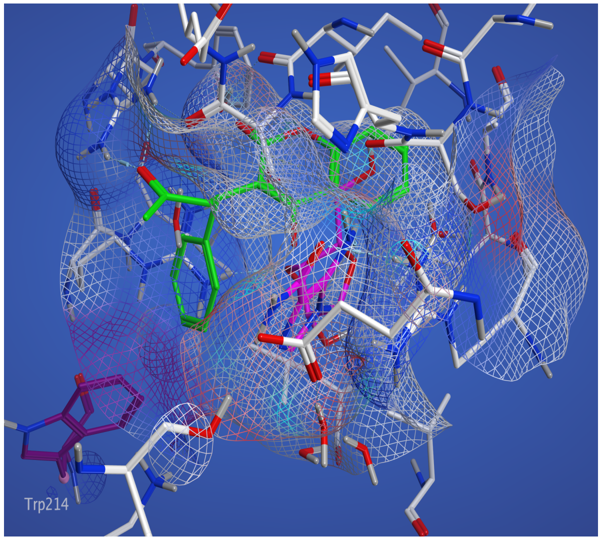


Figure 5c. HSA (PDB:ID 1N5U) bound

AICAR Sudlow site-I

Binding pocket VDW interaction Surface map

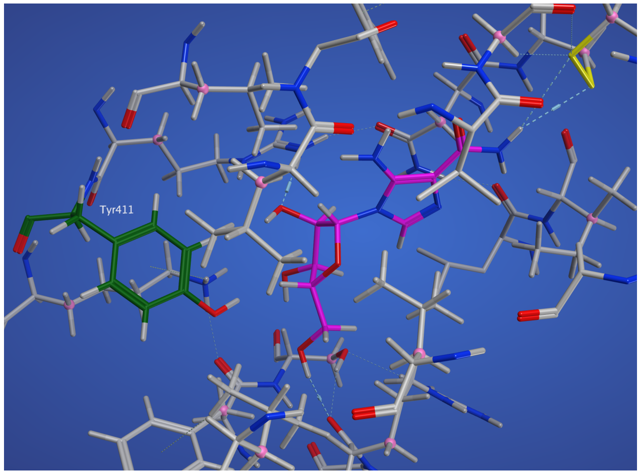


Figure 6a. HSA (PDB:ID 2BXG) bound

AICAR Sudlow site-II

AICAR (MAGENTA)

Tyrosine 411 (GREEN)

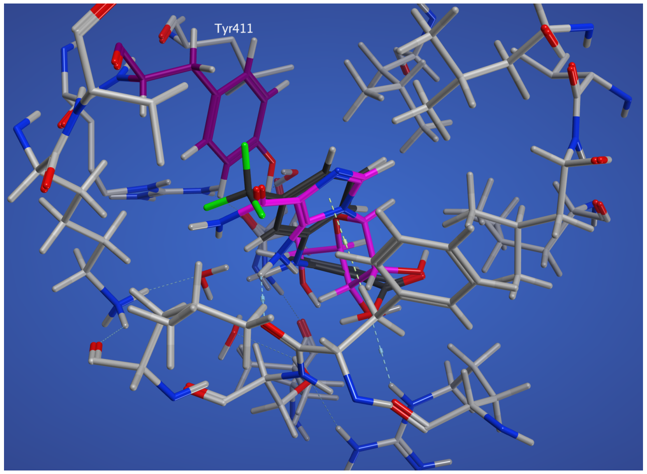


Figure 6b. HSA (PDB:ID 1N5U) bound

AICAR Sudlow site-II

AICAR (MAGENTA)

Flufenamic acid (black) Tyrosine 411 (purple)

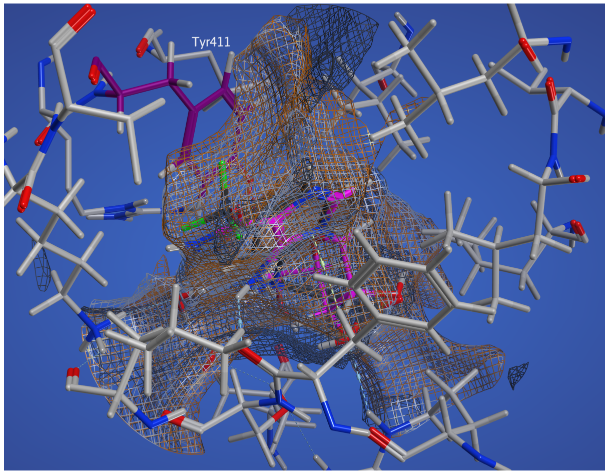


Figure 6c. HSA (PDB:ID 1N5U) bound

AICAR Sudlow site-II

Binding pocket VDW interaction Surface map

Figure 7: Ribbon Rendition of AICAR bound to HSA Site-I (PDB ID: 1N5U) AutoDock Vina, v.1.1.2

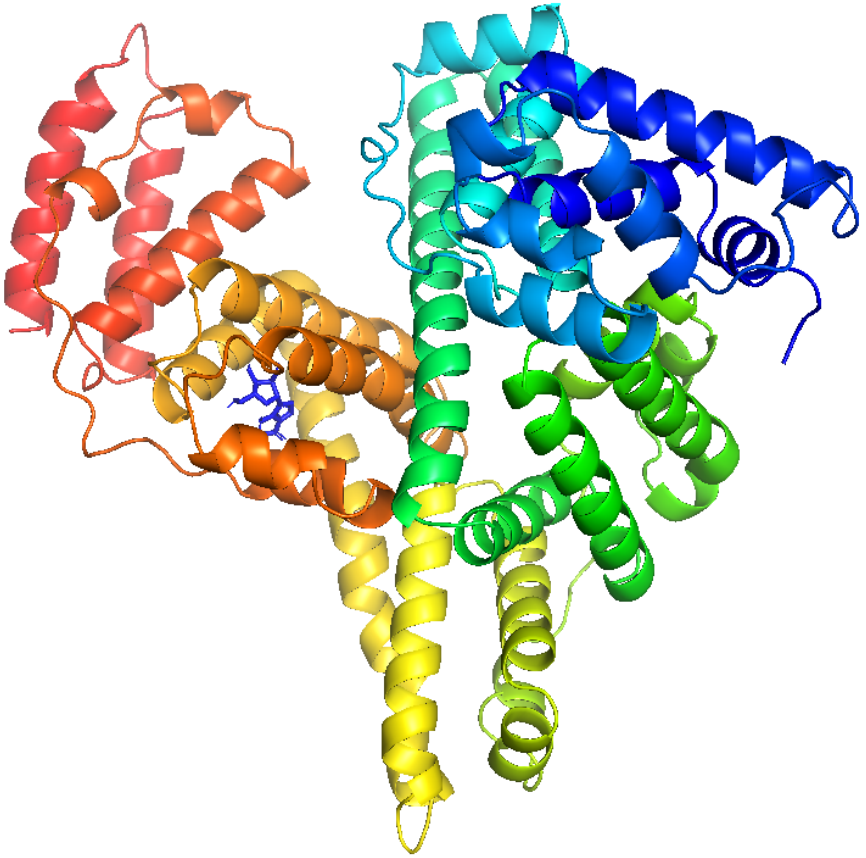

Figure 8: Ribbon Rendition of AICAR bound to HSA Site-II (PDB ID: 1N5U) AutoDock Vina, v.1.1.2

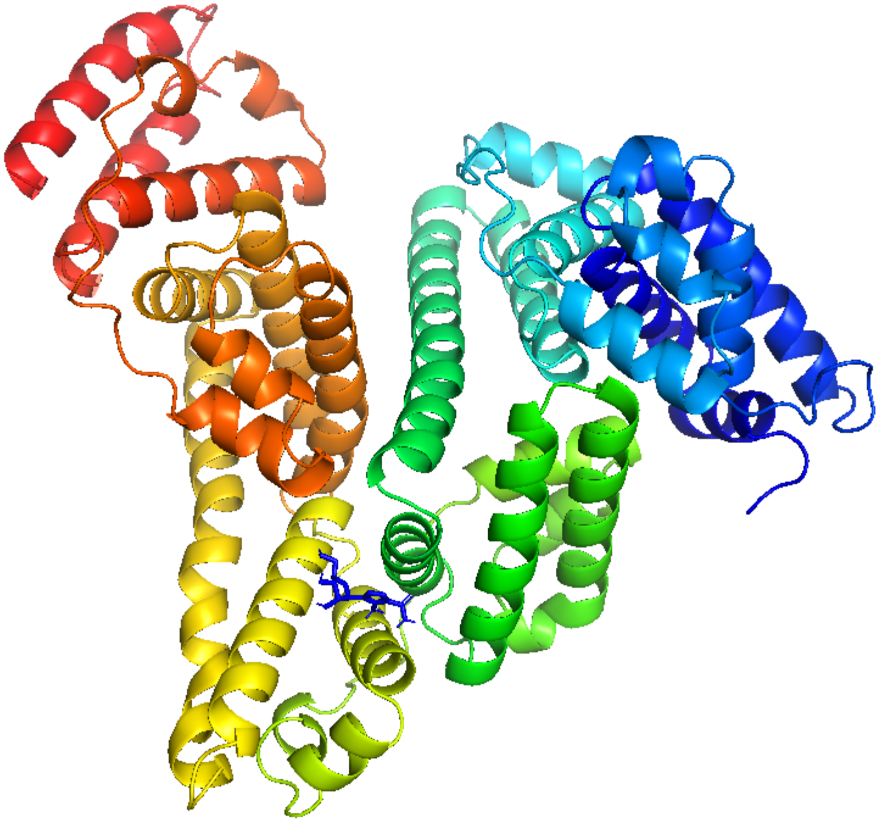

Supplement: Supplementary file 1 [file molecules-25-05410-s001.pdf]
